# The Week's Work

**Published:** 1911-05-13

**Authors:** 


### The Week's Work.

### WAR OFFICE METHODS AND THE PARKHURST INQUIRY.

There is little need to regret that the War Office inquiry into the alleged understaffing at Parkhurst Military Hospital was private, since its conclusions, whatever they may have been, were completely vitiated by sentence having apparently been pronounced before the inquiry was completed. Early last week the following order was issued from Portsmouth Headquarters: "Transfer of charge of Military Hospital: Major W. E. Hudleston will proceed to Parkhurst on Thursday, May 4, and assume charge of Military Hospital and command detachment R.A.M.C., vice Lieutenant-Colonel J. F. Donegan, who will proceed to the Alexandra Hospital, Cosham, for duty." The Court only concluded its labours on Thursday night. In other words, Lieutenant-Colonel Donegan was to be punished without having an opportunity of saying a word in his own defence, and to aggravate its action against him his superior officer at Cosham was to be an officer of his own rank, actually serving at the moment on the court of inquiry. Great indignation has been excited at the arbitrary action of the authorities in condemning a man before anything has been proved against him, and it is greatly to be hoped that further questions in Parliament as to who is responsible for this injustice will be put and pressed, Mr. Acland's answers on Tuesday being quite evasive. Lieutenant-Colonel Donegan has a record of service that in any other department of human activity except the Royal Army Medical Corps, would excite respect and consideration. Apart from his training in the two professions of medicine and the Army, he has served with distinction in two Burmese wars, on the North-West Frontier of India, and in South Africa, and has obtained official confirmation of his services by the bestowal of medals and mention in despatches. The public, and the men under him at Pankhurst, will certainly add to this list his repeated reports on the paucity of his staff, which, in spite of Mr. Acland's denials, are widely believed to have led to his present treatment.

### AN OFFICIAL EXAMPLE OF HOW NOT TO DO IT.

Let us summarise the facts already made known in the daily newspapers. The Southern Command is only some eight years old, and has instituted a system of red-tape by which every document has to be copied three times. The late Sergeant Stokes, as ward-master, steward, and dispenser had to work for some sixteen hours a day, and the system of documentary duplication already mentioned demanded of him the powers and time of a trained clerk into the bargain. Lieutenant-Colonel Donegan reported that one man's whole time was required for this purpose alone. He asked that a private should be appointed, who had been technically trained in the Surgeon-General's office, and whose pay would be 1s. 11d. per day. In the reply from the authorities Lieutenant-Colonel Donegan was told "to employ the corporal-cook in his spare time." That is the policy which directs the administration of the Royal Army Medical Corps. It has been followed by the death of Sergeant Stokes, by the appointment of a Court over whose heads sentence was pronounced while they were actually sitting, and now by the not-surprising lapse of Lieutenant-Colonel Donegan on to the sick list. It is no exaggeration to say that Lieutenant-Colonel Donegan has been treated with every aggravation into which official incapacity could blunder. The transfer, which itself reflects on him, has not merely relieved him from duty, so that a favourable issue from the inquiry would leave him as before; it has injured his status by placing him under an officer of his own rank, and, as a last practical injury, has threatened his pocket by making probable an exit from his house so hurried as to make him sacrifice the remainder of his tenancy. Military discipline and justice have produced many famous examples of how not to do it in their time; Denshawai is not forgotten; but the case of Sergeant Stokes is at our own doors, and public opinion is not too lately informed of the facts to secure the revision of the "sentence" against his helper and superior officer. As a last word, let it be remembered that a favourable report from the court of inquiry will only aggravate the arbitrary injustice of what has been done.

### AN INTERESTING TYPE OF COMPLETE WARD UNIT.

The instalment of the above series which we publish this week is particularly interesting, since it provides for a larger ward accommodation than in the preceding paper. The conception is that of Mr. Edwin T. Hall, who is a Fellow of the Royal Institute of British Architects, a Fellow and Member of the Council of the Royal Sanitary Institute, the Chairman of the Dulwich Estate Governors, and late President of the Architectural and Engineering Congress at Bristol in 1906. As a hospital architect Mr. Hall is well known. He is one of the architects to the Metropolitan Asylums Board, architect to the British Home and Hospital for Incurables, the Royal Naval School, and the Camberwell Guardians. He is responsible for the plans of the Park Hospital, Hither Green, the new Plaistow Hospital, the Seacroft and Killingbeck Hospitals, Leeds, the Camberwell Infirmary, and the Funeley Sanatorium, and was joint architect of the fine Manchester Infirmary, which is one of the most interesting models in this country. His experience in, and knowledge of, hospital construction entitle his opinions to respectful consideration, but do not necessarily exempt them from criticism, and we shall be glad to hear our readers' views on the designs he has contributed to the discussion.

### AN ADDITION TO CARDIFF INFIRMARY.

A great success was made last Saturday of the opening of the new gynæcological operating theatre by Lady Aberdare, whose generosity and persuasion have been recognised by the christening of the theatre with her name. Sir Francis Champneys, chairman of the Central Midwives Board, delivered an address in which he remarked on the value to the infirmary of lying-in wards, and said that "a woman was a very real state official when she had a family to look after." Sir Francis was followed by Colonel Bruce Vaughan, who said that £49,000 had been spent on the new wing, which contains eighty-two beds, and £62,000 invested for income, showing thereby the heroic efforts which he and Mr. Leonard Rea, the secretary, have made to obtain proper recognition for the work of the principal hospital of Wales. The year 1910-11 has been a wonderful year for the successful launching of appeals, and the conduct of that for Cardiff Infirmary has shown that it is not in London or England only that these can be conducted with statesmanship. It is noteworthy that the support which this institution has evoked has been obtained largely by intelligent argument and illustration, showing that these methods in capable hands are not inferior to the entertainment system.

### HOSPITALS AND FLANNELETTE.

In our correspondence columns appears a letter dealing with avoidable accidents due to the use of flannelette clothing. The inflammability of flannelette is well known, and the danger underlying its use has been pointed out more than once in our pages, especially when we referred to the findings of the various commissions appointed to investigate the question of prevention of deaths due to accidents. Our correspondent complains that hospital authorities indirectly encourage the use of flannelette by patronising flannelette manufacturers and by not insisting that all the cloth should be fireproofed. In our experience this is not quite correct. We know of no London hospital in which flannelette is largely used; where it is employed at all care is usually taken that the cloth is fireproofed by one or other of the methods which are generally considered reliable. As a matter of fact hospital authorities in the wards and in the out-patient department discourage the use of flannelette as much as possible. They see too many fatalities resulting from such use to pose as defenders of a material whose only good point is its relative cheapness.

### LADIES AND THE BOARD AT BEDFORD.

At the recent annual meeting of the Bedford County Hospital there were eight vacancies on the Board of Management and nine candidates, one of whom was a lady, Miss Stacey. While the voting was proceeding Lord Ampthill interposed to ask whether the nomination of a lady was held by the governors to be against the understanding arrived at in 1907, when two ladies were elected to the board. In his opinion they did not bind themselves on that occasion to the election of two ladies only. He asked in order that the point might be decided before instead of after the election. Lord Ampthill then enlarged upon the valuable help that had been given by the two ladies who had sat since 1907, and said that the idea was now that the number of ladies on the board should be limited not by their right, but merely by their popularity and influence. After a good deal of discussion the meeting agreed to Lord Ampthill's principle, but on the result of the voting being taken Miss Stacey was found to have obtained the smallest number of crosses. She can, however, find consolation in the fact that the governors who failed to elect her have admitted her right to oust them from the board if she can.

### THE RELATION OF THE PAY WARD TO THE NURSING HOME.

One of the objections systematically brought against the general installation of pay wards at the voluntary hospitals is the commercial one that they bring the institution which adopts them into unfair competition with the nursing home. The supporters of pay wards, on the other hand, of whom the Bishop of Birmingham is an energetic lay example, urge that pay wards exist for a class which the nursing home cannot hope to reach—a class, in other words, for which two guineas is the maximum fee. The question is complicated for hospitals, however, by the fact that the presence of pay wards misleads the public into supposing that they are run at a profit, and thereby reduces the subscription list on the ground that the hospital is largely a commercial institution. This misconception has led several hospitals which have installed pay wards into charging fees that at any rate safeguard them against loss, and of such fees three guineas a week is the minimum. In any case the smooth working of the pay-ward system demands that the patients should be attended, if they desire it, by their own practitioners, and should not be limited to the hospital's staff. It is not unknown for a hospital to set all the local practitioners against it by not conceding the right of the paying patient to be attended by his own doctor. All the practitioners in the district should have the right to send their patients to the pay wards. If this is not conceded they will send their own patients anywhere rather than run the risk of losing them to members of the hospital's medical staff. It is sometimes said that hospitals should not erect pay pavilions, on the ground that it has no right to risk charitable funds. If this argument holds, no pavilions should be erected at all. All wards in a voluntary hospital are run, and are intended to be run, at a loss. That is the essence of the voluntary system. Wards for paying patients at a voluntary hospital are intended to be run at a little less loss than other wards, otherwise there is no charity. The moment they show a profit the commercial argument of unfair competition with the nursing home cannot be met.

### THIS WEEK'S DRUG MARKET.

The drug market has been rather more active during the week, but there is still room for much improvement. Cod-liver oil has again declined in value, and if the demand does not improve prices may recede still further; the recent temperate weather has not tended to increase the consumption of this oil, and in some degree this accounts for the fall in value. The price of opium is well maintained. Ipecacuanha is slightly dearer, as also is Cape aloes. Jamaica honey fetched much higher prices at the recent drug sales. Other drugs which have advanced in price are sarsaparilla, hydrastis, canadensis, and pepsin.

